# Effect of Addition Amount of Ethylenediamine on Interlayer Nanochannels and the Separation Performance of Graphene Oxide Membranes

**DOI:** 10.3390/polym16223123

**Published:** 2024-11-08

**Authors:** Na Meng, Xin Sun, Jinxin Liu, Jialing Mi, Rong Rong

**Affiliations:** Jiangsu Key Laboratory of Industrial Pollution Control and Resource Reuse, School of Environment Engineering, Xuzhou University of Technology, Xuzhou 221018, China; sunxin2708@163.com (X.S.); ljx2667495913@163.com (J.L.); 17751282269@163.com (J.M.); rongrong114717@163.com (R.R.)

**Keywords:** graphene oxide, D-spacing, crosslinking, EDA, destaining and desalting

## Abstract

In recent years, graphene oxide (GO)-based two-dimensional (2D) laminar membranes have attracted considerable attention because of their unique well-defined nanochannels and deliver a wide range of molecular separation properties and fundamentals. However, the practical application of 2D GO layered membranes suffers from instability in aqueous solutions as the interlayer *d*-spacing of GO membranes is prone to expansion caused by the hydration effect. In this study, the effects of the ethylenediamine (EDA) addition amount on the structure, crosslinking mechanism and separation performance of GO membranes were investigated systematically, and membrane performance was evaluated using water permeability and dye/salt rejection tests. The experimental results show that the amine groups of EDA chemically bond with the hydroxyl functional group (O=C–OH) of GO after intercalation, as evident from Fourier transform infrared spectroscopy (FTIR) and X-ray photoelectron spectroscopy (XPS). By further controlling the amount of the intercalated EDA, the as-prepared GO composite membranes show nanoscale-tuned *d*-spacing promising for downstream applications. In the demonstrated dye/salt nanofiltration scenario, the EDA intercalated and crosslinked GO membrane has enhanced permeability by over five times and a better dye rejection rate of over 96% compared with pure GO membranes. These findings highlight a facile strategy for controlling nanochannels by tuning the amounts of reactive intercalants.

## 1. Introduction

Nanofiltration membrane technology, which has developed rapidly over the past few years, shows significant advantages in the fields of seawater desalination, molecular separation and water filtration [1,2,3]. With a small pore size, nanofiltration membranes selectively screen separators mainly through sieve size and charge effect on membrane surfaces [4]. Meanwhile, they also show a good ability to remove the chrominance in water, which indicates their potential to treat dyes and desalting [5,6].

As a classic two-dimensional (2D) material, graphene oxide (GO) is abundant in oxygen-containing groups (carboxyl, hydroxyl and epoxide) on its surface and edge [7,8,9], which endows it with strong hydrophilicity and pollution resistance [10,11]. In addition, the membranes prepared by GO have a unique layered structure and nanoscale channels and are suitable for size-dependent molecular screening and water penetration. These properties make GO membranes suitable alternatives for conventional polymeric nanofiltration membranes [12,13,14,15,16,17]. However, the performance of GO membranes is affected by the solution diffusion mechanism [18]. When a GO membrane is placed in an aqueous solution, water molecules will first dissolve in the membrane material and diffuse through the membrane. This leads to the insertion of two–three layers of water molecules into the interlayer of GO channels, which increases the spacing between GO neighboring layers and thus the phenomenon of “expansion”. This may give rise to the swelling of the layered structure of GO nanomembranes and destroy nanomass transfer channels, which thus limits the application of GO membranes in aqueous solutions [19,20]. 

To build a stable interlayer nanochannel of GO membranes and optimize the separation performance of membranes, some methods have also been tried to prepare GO membranes. At present, the main preparation methods include crosslinking, partial reduction and multi-material hybrid methods [21]. In general, the crosslinking method is employed to prepare GO membranes by connecting GO nanosheets with crosslinking agents to inhibit the hydration of GO membranes and improve their stability [22]. Commonly used crosslinkers include molecular polymers containing amino groups, such as ethylenediamine (EDA) [23], p-phenylenediamine [24] and polyethylenimine [25]. The amines contained in these polymers can be condensed with the carboxyl groups on the GO to produce a new C-N covalent bond to fix the GO membrane. For example, Hung et al. [23] selected three diamine monomers (EDA, butylenediamine and p-phenylenediamine) and successfully prepared GO composite nanofiltration membranes with different layer spacings via pressure-assisted self-assembly technology. Thus, the layer spacing stretching caused by membrane expansion was effectively inhibited. Wansuk et al. [26] coated GO multilayer membranes on the surface of polyamide membrane composite membranes by depositing GO nanosheets with opposite charges layer by layer. The results show that the prepared membranes have excellent anti-fouling and -chlorine properties. 

In addition to using amine molecules with different structures to build interfacial molecular bridges to improve membrane performance, it has also become a direction to optimize the performance of GO membranes by embedding crosslinker molecules with different qualities. By adjusting the molecular weight of polyethyleneimine (PEI), Wang et al. [27] fine-tuned the interlayer structure, surface charge and hydrophilicity of a GO membrane, and the optimized membrane showed a high desalination rate and excellent mechanical stability. Kong et al. [28] found that the addition of more dopamine (DA) can weaken the hydration of GO nanosheets and overcome swelling in water, which effectively adjusts the interlayer spacing and crosslinking network structure. As a result, GO membranes can still maintain the performance of accurate separation in aqueous solutions. In previous studies [23,29,30], EDA has been reported to be an effective crosslinking agent for GO nanosheets, and EDA crosslinked GO composite membranes have lower interlayer spacing compared to other diamine monomers. Nevertheless, few studies have examined the effect of the amount of EDA on the material transport and interlayer structure of GO membranes. This research gap prompted researchers to systematically study the mass transfer process of GO membranes by adjusting different EDA amounts. This study aimed to study the feasibility and mechanism of EDA mediating GO membranes on nanofiltration structure and sieving performance by regulating the EDA amount. 

In this study, a simple vacuum filtration method was used for synthesizing stable mixed cellulose ester (MCE)/GO/EDA membranes. By adding different amounts of EDA, an interfacial molecular bridge was constructed to regulate the layer spacing and screening performance of GO composite nanofiltration membranes. The structure and screening performance of EDA-mediated GO membranes were further studied.

## 2. Experimental Section

### 2.1. Materials

Single-layer graphene oxide (GO, Jiangsu Xianfeng Nanomaterials Technology Co., Ltd., Nanjing, China). Pure sodium chloride (NaCl, Sinopharm Chemical Reagent Co., Ltd., Shanghai, China), sodium sulfate (Na_2_SO_4_, Sinopharm Chemical Reagent Co., Ltd., Shanghai, China) and magnesium sulfate (MgSO_4_, Sinopharm Chemical Reagent Co., Ltd., Shanghai, China). Ethylenediamine (EDA, Shanghai Maclin Biochemical Technology Co., Ltd., Shanghai, China). Hybrid Cellulose Ester Microporous Filter Membrane (MCE, Hangzhou Micropay Technology Co., Hangzhou, China) with a pore size of 0.22 μm. Methylene Blue (MB, Shanghai Aladdin Biochemical Technology Co., Ltd., Shanghai, China). The experimental water used was deionized water.

### 2.2. Preparation of MCE/GO/EDA Composite Membranes

**Figure 1:**
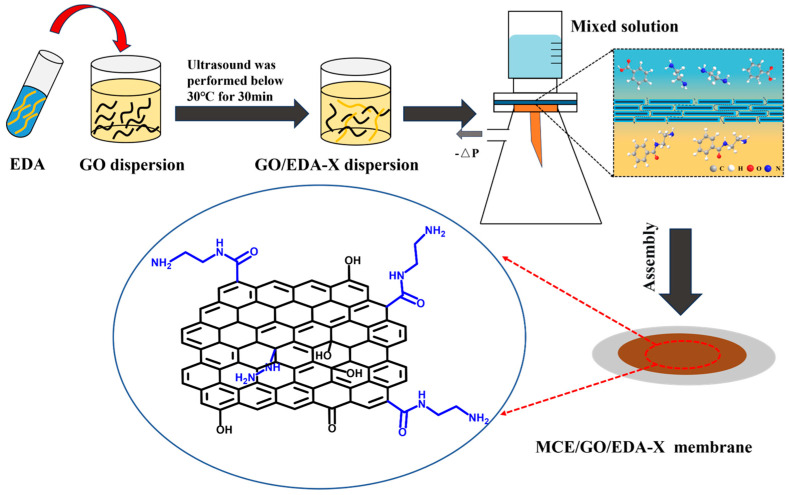
Preparation process of MCE/GO/EDA composite membranes.

After 20 mg of GO powder was weighed and mixed with 500 mL of deionized water, 40 mg/L of GO dispersion was obtained by ultrasound at 30 °C for 30 min. After 40 mL and 40 mg/L of GO dispersion were evenly mixed with 100 mL of deionized water, different amounts of EDA were added, and the GO/EDA mixture was made using ultrasound at 30 °C for 30 min. Then, the mixture was filtered to the MCE membrane through a vacuum filtration device by the vacuum filtration method to obtain the MCE/GO/EDA membrane. The different types of MCE/GO/EDA membranes are named according to the amount of EDA added. For instance, MCE/GO/EDA-X indicates that the amount of EDA added to the graphene oxide composite membrane is “X” mL. Figure 1 shows the preparation process of the MCE/GO/EDA composite membrane.

### 2.3. Characterization of MCE/GO/EDA Membranes

Fourier transform infrared spectroscopy (FTIR, Nicolet iS10, Thermo Fisher Scientific, Waltham, MA, USA) and X-ray photoelectron spectroscopy (XPS, Thermo Scientific K-Alpha, Thermo Fisher Scientific Waltham, MA, USA) were utilized for analyzing the functional groups and structures of MCE/GO/EDA membranes. The surface and cross-section morphology of MCE/GO/EDA membranes were observed by scanning electron microscopy (SEM, Regulus 8100, Hitachi, Japan). The crystal structure of GO nanosheets was analyzed using an X-ray diffractometer (XRD, D8 ADVANCE, Bruker, Germany). The sample was scanned from 5 to 55°. The d-spacing (d) of the prepared GO membrane can be calculated by Bragg’s law in Equation (1).

(1) $$d=\frac{n\lambda }{2sin\theta }$$

where λ is X-ray wavelength; θ is the diffraction angle; and n is the diffraction order.

The hydrophilicity of the surface of the prepared membrane materials was tested using a contact angle tester (CA, JY-82C, China Chengde Dingsheng, China) at least twice for each membrane. The surface structure and roughness of membranes were observed using atomic force microscopy (AFM, Bruker Dimension ICON, Bruker, Germany). The surface charge of the GO membrane was observed using a zeta potential analyzer (Anton Paar surpass3, Anton Paar Austria Ltd., Graz, Austria).

### 2.4. Evaluation of Membrane Performance

The permeability of MCE/GO/EDA membranes was assessed by measuring membrane flux using deionized water as the feed solution. NaCl, Na_2_SO_4_, MgSO_4_ and 10 ppm MB solutions with concentrations of 1000 ppm were used as feed solutions to test the rejection performance of membranes.

A dead-end filtration device was applied to test membrane performance, and a diagram of the device can be obtained from our previous study [31]. The filtration system consists of a nitrogen pressurizer, a sealable water tank, a digital balance, a 300 mL filter cup, a magnetic stirrer and computerized online measurement software. Regarding the measurement of rejection performance, the specific filtration process is as follows: (1) the membrane was compacted first for 2 h to achieve a stable flux, with a transmembrane pressure of 2 bar; (2) the transmembrane pressure was reduced to 1 bar, and the pure water flux was recorded every 30 s. At least 40 measurements were collected to obtain average flux values; (3) deionized water was replaced by salt and MB feed solution. Filter cups were stirred using a stirring rod at 600 rpm to minimize concentration polarization, and then the transmembrane pressure was restored to 1 bar. After filtering for a period of time, 15 mL of filtrate was gathered. 

Membrane permeability (J, Lm^−2^ h^−1^ bar^−1^) was determined according to Equation (2) by membrane effective area (A, m^2^) per unit time (T, h) per unit pressure (P, bar) per volume of penetrant (V, L).

(2) $$J=\frac{V}{T\times A\times P}$$

The conductivity meter was utilized to measure the conductivity of the filtrate and stock solution before filtration to evaluate the desalting performance of the membrane. The dye solution was measured using ultraviolet–visible spectrophotometry (UV-VIS) to evaluate the effect of the membrane on dye rejection. 

The rejection of salt ions and dyes (R) was calculated using Equation (3). C_0_ and C_1_ corresponded to the concentrations of the feed solution and filtrate, respectively.

(3) $$R=\frac{C_{0}-C_{1}}{C_{0}}\times 100\%$$

## 3. Results and Discussion

### 3.1. FTIR Characterization of MCE/GO/EDA Membranes

**Figure 2:**
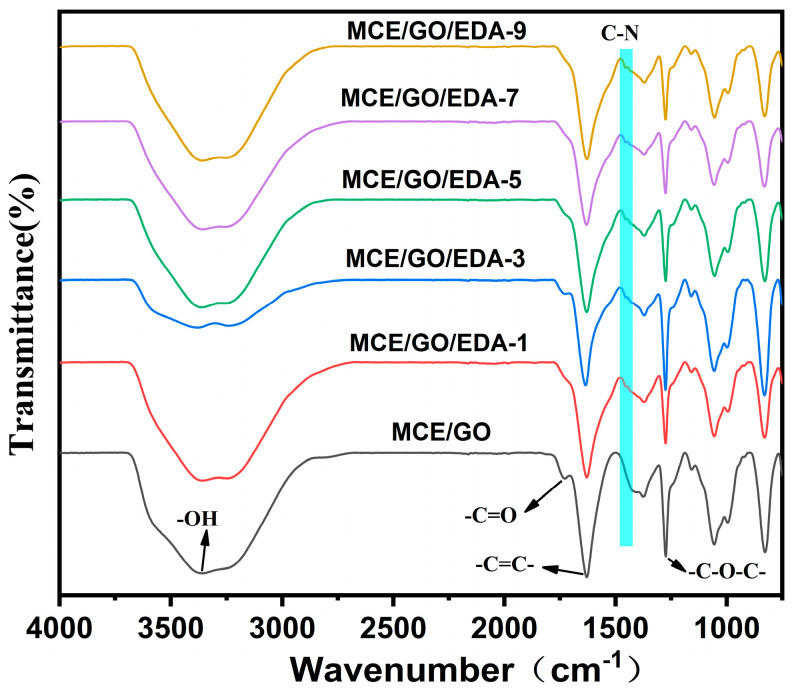
Fourier infrared spectra of MCE/GO and MCE/GO/EDA-X membranes.

The membrane was characterized by FTIR to illustrate the bond between EDA and GO. As shown in Figure 2, the original GO membrane is rich in oxygen-containing groups, among which absorption peaks can be observed at 3352, 1730, 1631 and 1274 cm^−1^ wave numbers. Corresponding to hydroxyl and carboxyl groups (–OH and –C=O), carbon–carbon double bond (–C=C–) and epoxy group (–C–O–C–), these data are consistent with the research results of Chen et al. [32]. After EDA treatment, the carboxyl group (–C=O) disappeared in the MCE/GO/EDA membrane; hydroxyl and epoxy groups (–OH and –C–O–C–) significantly decreased; and a new absorption peak (C–N, 1454 cm^−1^) appeared, which indicated that the amine group of EDA would condense with the carboxyl group of GO. The nucleophilic substitution reaction with the epoxide group formed C–N covalent bonds [23], and the crosslinking reaction occurred successfully. Due to relatively mild crosslinking reaction conditions during the preparation of the composite membrane, the new absorption peaks generated were not significant. Hence, XPS was further used to analyze the composition of elements in the membrane and understand the reaction principle of GO and EDA. 

### 3.2. XPS Characterization of MCE/GO/EDA Membranes

XPS was used to further analyze the crosslinking reaction between GO and EDA. As presented in Table 1, the increase in EDA amount from 1 to 9 mL was accompanied by a decrease in the O element of the EDA-loaded GO membrane from 27.1% to 21.2%, an increase in the N element from 4.79% to 5.74%, and a decrease in the O/C ratio from 0.40 to 0.29. The ratio (0.29) of O element to C element in the EDA-loaded GO membrane is significantly lower than that (0.49) of O element to C element in the original MCE/GO membrane. This is due to the reaction of EDA with the oxygen-containing functional groups of GO and the loss of oxygen-containing functional groups in the reaction process, which reduced the O/C ratio [33]. 

**Figure 3:**
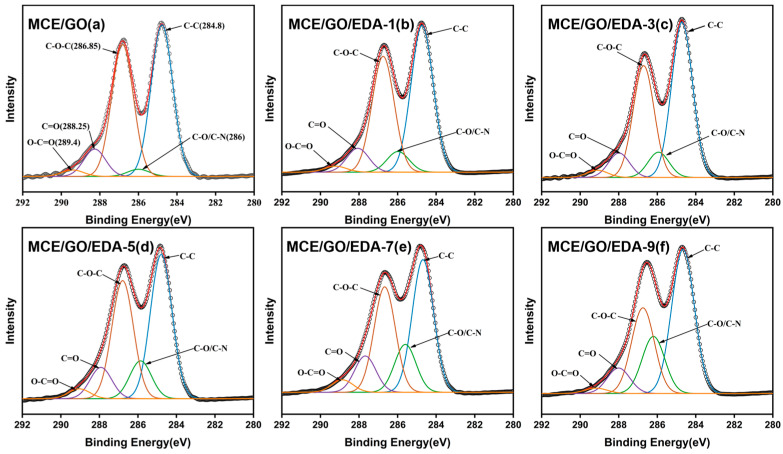
Analysis of XPS elements in the C1s region of the original MCE/GO membrane (**a**) and MCE/GO/EDA composite membrane (**b**–**f**).

It can be seen from Figure 3 that the scanning spectra of the XPS C1s region of the original MCE/GO membrane and MCE/GO/EDA composite membrane with different amounts of EDA were decomposed into five peaks: C–C (284.8 eV), C–O/C–N (286 eV), C–O–C (286.85 eV), C=O (288.25 eV) and O–C=O (289.4 eV). Compared with the original MCE/GO membrane, the modified MCE/GO/EDA membrane exhibited a decrease in the C–O–C peak and an increase in the C–O/C–N peak. This is because GO contains active epoxy groups, and its exposure to the amine group leads to the active ternary epoxy ring opening reaction, which results in a new C–N bond [34]. This confirms that the crosslinking reaction between EDA and GO is successful. As illustrated in Figure 3b–f, the C–O–C peak of the MCE/GO/EDA membrane decreased, and the C–O/C–N peak increased more significantly with the increase in EDA amount. This also indicates that more oxygen-containing groups were consumed in the reaction to generate C–N covalent bonds, which greatly increased the stability of the membrane. 

### 3.3. Zeta Potential and Water CA Analysis

**Figure 4:**
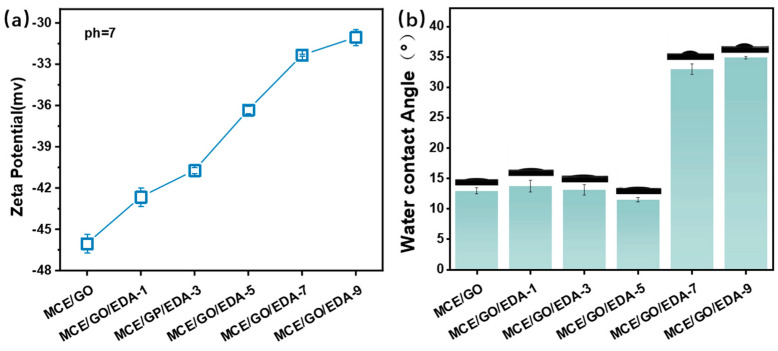
Surface zeta potentials (**a**) and water CAs (**b**) of MCE/GO and MCE/GO/EDA-X membranes.

To investigate the effect of the embedding amount of EDA on the surface charge of the GO membrane, the zeta potentials of MCE/GO and MCE/GO/EDA-X membrane samples at Pondus hydrogenii (pH) = 7 were measured. Previous reports have demonstrated that GO membranes are negatively charged due to numerous carboxyl and hydroxyl functional groups on the surface [35,36]. As shown in Figure 4a, the surface potential of the original MCE/GO membrane is −46.02 mv, which is highly consistent with the research report of Zhang et al. [37]. In the meantime, it can be observed that the electronegativity of the membrane surface began to weaken with the increase in the embedding amount of EDA, but it still had a negative charge. This evolution of surface charge may be attributed to the removal of oxygen-containing functional groups by a large amount of EDA reduction. This indicates that surface charge can be properly regulated by changing the embedding amount of small amine molecules, which would play an important role in ion transport in the membrane.

It can be observed from Figure 4b that the CA of the MCE/GO/EDA membrane generally presented a trend of decrease with the increase in the EDA addition amount from 0 to 5 mL. Additionally, the hydrophilicity of the MCE/GO/EDA membrane was improved, which was related to the abundant oxygen-containing hydroxyl groups (–OH) on the membrane. The epoxide group (–C–O–C–) was associated with the amine group (–N–H–) [28]. In addition, the change in the surface roughness of the membrane also changed its hydrophilicity [38,39]. However, the water CA of the MCE/GO/EDA membrane also began to increase, and its hydrophilicity decreased as the amount of EDA continued to increase. This indicates that the oxygen-containing groups on the surface of the MCE/GO/EDA membranes are reduced by EDA, and the hydrophilicity of the amines grafted onto the membranes is weaker compared to the hydrophilic hydroxyl groups, which greatly reduces the hydrophilicity of the membranes.

### 3.4. XRD Characterization of MCE/GO/EDA Membranes

**Figure 5:**
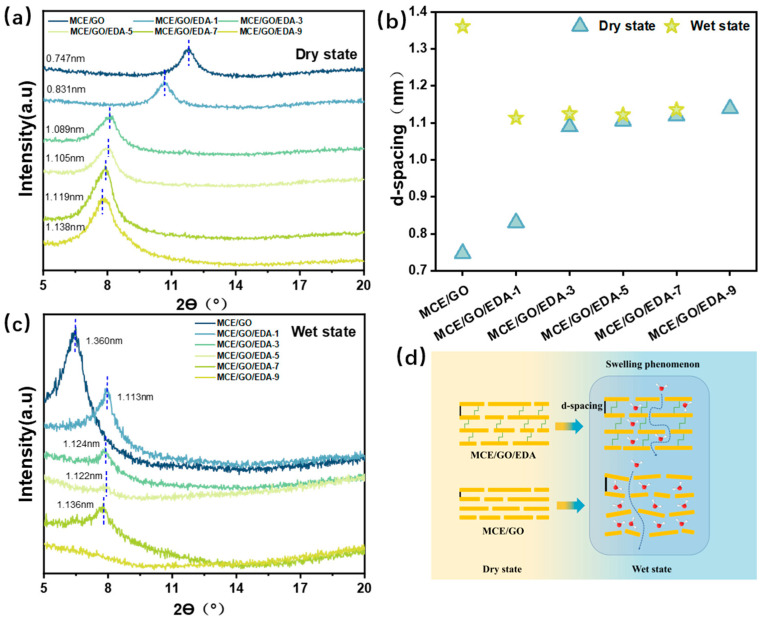
XRD patterns and d-spacing of MCE/GO and MCE/GO/EDA-X membranes: (**a**) dry state; (**b**) D-spacing; (**c**) wet state; (**d**) swelling phenomenon.

According to the XRD pattern of the composite membrane, the Bragg formula was used to calculate the layer spacing of the composite membrane mediated by EDA with different amounts in dry and wet states (Figure 5a,c). In the dry state, the d-spacing corresponding to the original GO membrane is 0.747 nm, which is basically in line with the previously reported results [16,33]. With the increase in EDA amount, the layer spacing of the MCE/GO/EDA membrane increased. This shows that membrane spacing can be adjusted by adding different EDA amounts to different substances, which is expected to achieve the effect of accurately removing ions and molecules of different sizes. In addition, it can be seen from the MCE/GO and MCE/GO/EDA membranes tested in the wet state that the original MCE/GO membrane significantly expanded the size of the interlayer nanochannel due to the widespread swelling phenomenon (Figure 5d). Water molecules filled the membrane interlayer and stretched the interlayer spacing, which would seriously weaken the ion exclusion ability of the GO membrane [40]. Of note, the structure of the MCE/GO/EDA membrane did not change significantly with the increase in the embedding amount of EDA (Figure 5b). This is because the amine group of EDA formed effective C–N covalent bonds within the GO membrane to interlock GO nanosheets, which resisted the d-spacing stretching phenomenon [41]. The layer spacing of the MCE/GO/EDA-9 membrane cannot be accurately measured, possibly because excessive crosslinking would increase the disorder of the membrane in the water environment. Consequently, the intensity of the XRD peak was not observed. 

### 3.5. SEM Characterization of MCE, MCE/EDA and MCE/GO/EDA Membranes

**Figure 6:**
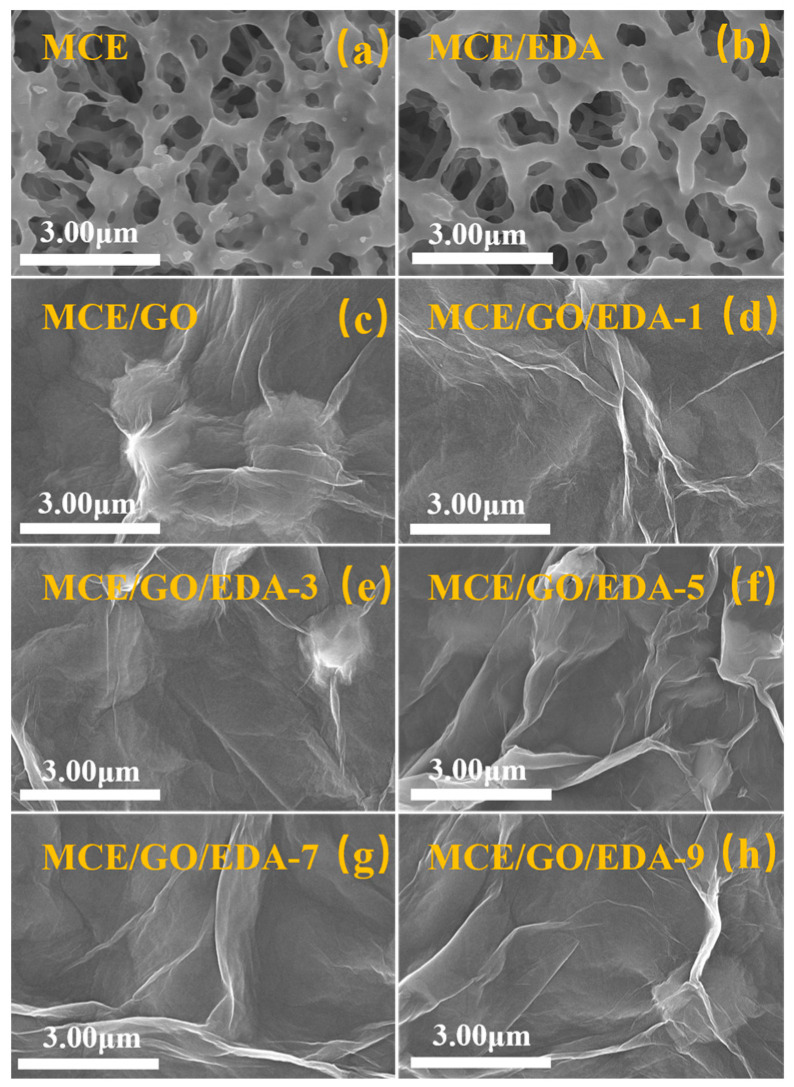
SEM surface images of MCE base (**a**), MCE/EDA (**b**), MCE/GO (**c**) and MCE/GO/EDA-X membranes (**d**–**h**).

**Figure 7:**
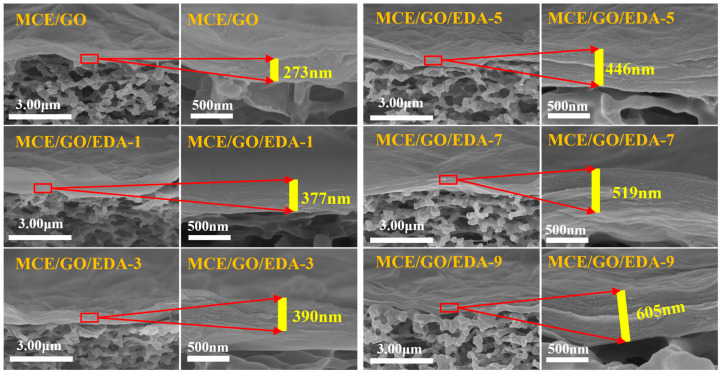
SEM cross-section images of MCE/GO and MCE/GO/EDA-X membranes.

The surface morphology and cross-sectional structure of the membrane were characterized by SEM (Figure 6 and Figure 7). MCE and MCE/EDA membranes were characterized for their comparison after EDA treatment. As shown in Figure 6a,b, the surface aperture of the MCE/EDA membrane after EDA treatment did not change significantly compared with that of the original MCE membrane, which indicated that EDA did not change the surface morphology of the MCE base membrane. The surface morphology of MCE/GO/EDA membranes synthesized under different EDA loading loads is shown in Figure 6c–h. The surface of all MCE base membranes is covered by a uniform layer of GO nanosheets, with typical folded surface morphology and well-layered layered structure [42] owing to rich oxygen-containing groups on the surface of GO, which makes it form nanoscale folds in GO nanosheets and provides mass transfer channels for water molecules [43].

As shown in Figure 7, no obvious gap existed between the GO layer and MCE base membrane in the MCE/GO/EDA membrane after EDA treatment compared with the MCE/EDA base membrane. This also indicates that GO and MCE membranes were closely combined through EDA crosslinking. Figure 7 shows the change in the thickness of the GO nanolayer under different EDA addition amounts, which is aligned with the expectation. With the increase in the EDA addition amount from 0 to 9 mL, the thickness of the GO/EDA layer increased from 273 to 605 nm. The change in the thickness of the GO layer meant that EDA embedded in the GO layer membrane could adjust the layer spacing of the GO layer membrane, which is consistent with the XRD test results. 

### 3.6. AFM Characterization of MCE/GO/EDA Membranes 

**Figure 8:**
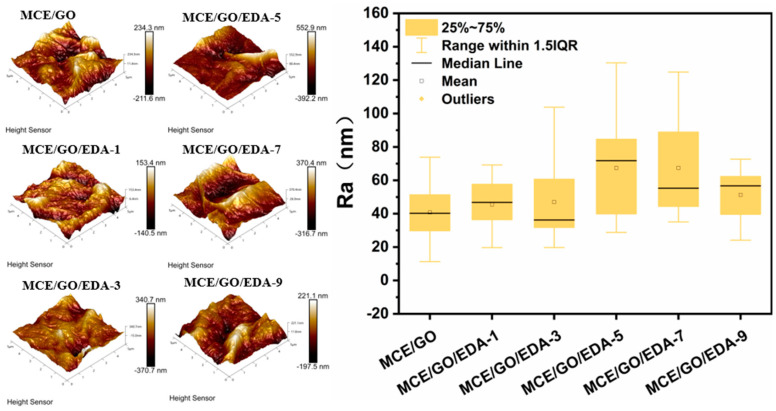
AFM images and surface roughness of MCE/GO and MCE/GO/EDA-X membranes.

The effect of EDA on the surface roughness of the MCE/GO membrane was further studied by using AFM to characterize surface morphology. The 3D AFM images of the surface of the MCE/GO/EDA membrane under different EDA addition amounts are shown in Figure 8. It can be seen that the roughness of the membrane surface increases and then decreases with the amount of EDA. It was confirmed that the variation in the roughness of the MCE/GO/EDA membrane was linked to the crosslinking reaction of EDA and GO, and a higher crosslinking degree would lead to a more uniform deposition of GO on membrane surfaces, which resulted in a decrease in roughness [28]. When EDA was embedded into GO interlayer nanochannels, the roughness of the MCE/GO/EDA membrane became larger. Rough membranes can provide more water contact sites, which is conducive to the rapid flow of water through membranes and the increase in water flux [44].

### 3.7. Salt Rejection Performance

**Figure 9:**
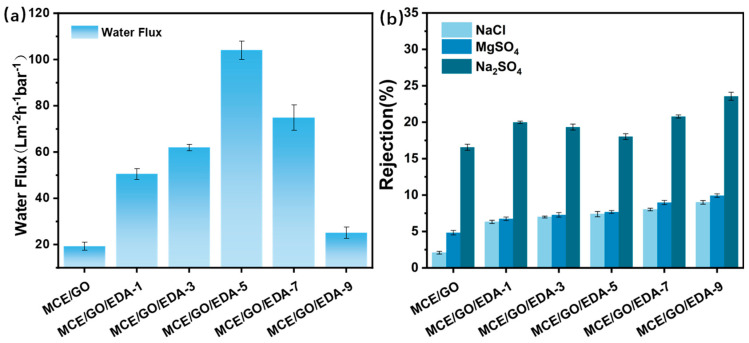
Effects of different EDA addition amounts on water flux (**a**) and salt rejection (**b**) of MCE/GO/EDA-X membranes.

In the research process, a 1 bar terminal filtration device was used to test the water permeability, desalting performance and dye removal performance of the MCE/GO/EDA membrane under different EDA loads. As shown in Figure 9a, the MCE/GO membrane without EDA treatment exhibited low water flux, which resulted in the collapse of interlayer nanochannels because the GO membrane was easy to compact [45]. In general, it can be seen that EDA addition has a great influence on the water flux of the MCE/GO/EDA membrane. When the load value of EDA increased from 0 to 5 mL, the pure water flux of the MCE/GO/EDA membrane presented an increasing trend, which may be due to the increase in GO layer spacing and the expansion of interlayer nanochannels mediated by EDA into the GO layer. The rapid passage of water molecules was facilitated through the GO nanolayer. Nonetheless, the water flux of the MCE/GO/EDA membrane was reduced when the amount of EDA was further increased to 9 mL because of excessive EDA mediating the GO layer, which could easily cause a “blockage” phenomenon, thus hindering the flow of water molecules. 

As shown in Figure 9b, the salt rejection rate of the GO membrane is Na_2_SO_4_ > MgSO_4_ > NaCl, and the GO membrane has a higher rejection rate of Na_2_SO_4_ than NaCl. The reason is that high-valence co-ions need to overcome a larger interaction energy barrier than low-valence ones during ion transport [46]. Mg^2+^ transmission is inhibited, and MgSO_4_ has a higher interception rate than NaCl since the hydration radius of Mg^2+^ is larger than that of Na^+^. Moreover, the rejection rate of Na_2_SO_4_ is higher than that of MgSO_4_, which concurs with previous research reports [47]. It can be observed from the figure that the rejection rate of Na_2_SO_4_ decreased slightly when the amount of EDA increased from 1 to 5 mL and began to rise when the amount of EDA increased to 9 mL. This is because excessive EDA made the structure of GO more dense [13], and the “blockage” phenomenon of interlayer nanochannels occurred. The rejection rate of Na_2_SO_4_ increased to 23.57%. By controlling the amount of EDA added, the layer spacing of the GO membrane can be adjusted, which thus affects its water flux and salt rejection performance. As demonstrated in Figure 9, the flux of the MCE/GO/EDA membrane reached the maximum value of 104.07 L/m^2^·h·bar with the addition of 5 mL EDA, while the salt rejection rate decreased. 

### 3.8. Dye Rejection Performance

**Figure 10:**
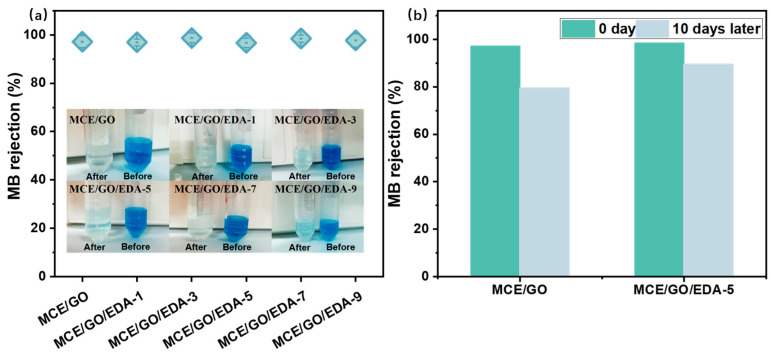
Effects of different EDA addition amounts on the MB dye removal (**a**) and stability (**b**) of the MCE/GO/EDA-X membrane.

It is well known that the dye removal effect of nanofiltration membranes is closely related to the pore size of membranes and the charge property of membrane surfaces [48,49,50]. As illustrated in Figure 10a, the removal rate of MB dye for MCE/GO/EDA membranes with different EDA addition amounts is above 96%. This indicates that the negative electronegativity of the membrane surface is weakened by the embedding of EDA into the GO membrane, which reduces the attraction with the positively charged methylene blue molecules and attenuates the permeation of methylene blue molecules within the membrane. Despite the increase in interlayer nano-channels, the high rejection rate of MB was maintained.

Zhang et al. [33] prepared GO-UR/CA membranes by crosslinking graphene oxide (GO) with urea (UR), and the rejection rates of KCl, NaCl and MgSO_4_ were 21.6%, 26.8% and 63.2%, respectively. Yuan et al. [51] used the covalent crosslinking of thiourea (TU) molecules with graphene oxide, which significantly increased the salt rejection rate (the rejection rate of NaCl was 95.6% and 90.2% for MgCl_2_). Chandio et al. [52] prepared cGO/SAA membranes using serine amino acid (SAA)-modified GO with excellent separation efficiencies of 99% and 98% rejection of rhodamine B and methylene blue, respectively. Compared with previous studies, the composite membranes prepared by crosslinking EDA with GO in this paper showed lower rejection of salt ions but had relatively high water flux and high dye rejection, which can help to achieve dye/salt selective separation in practical applications. 

To assess the stability of the membrane, it was immersed in a dye solution for 10 d, and the dye retention recovery rates of MCE/GO and MCE/GO/EDA-5 membranes were subsequently evaluated. As shown in Figure 10b, the initial dye retention rates of MCE/GO and MCE/GO/EDA-5 membranes were similar, and the dye retention recovery rate of the MCE/GO/EDA-5 membrane was 90.90% after 10 d of immersion, while that of the MCE/GO membrane was only 81.82% of the original value. This indicates that the MCE/GO/EDA-5 membrane has stronger stability than the MCE/GO one. 

**Figure 11:**
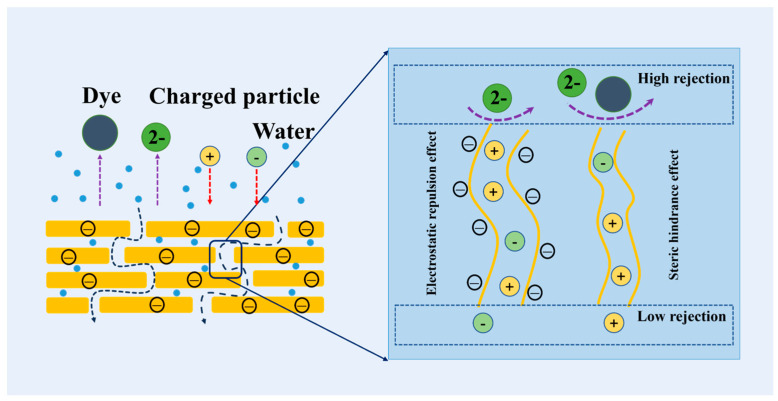
Ion transport mechanism of the MCE/GO/EDA-X membrane.

The desalting and dye removal performance of the MCE/GO/EDA membrane was mainly related to the steric hindrance effect and electrostatic interaction [4]. As shown in Figure 11, the negatively charged ions would be repelled by electrostatic repulsion and could not penetrate the GO membrane because the membrane was negatively charged. Meanwhile, some ions whose hydration radius is larger than the aperture were also blocked outside the membrane. For the mass transport in pores, larger molecules or ions have a higher affinity for interlayer nanochannels owing to their weak interaction with bound water molecules, which resulted in the phenomenon of adhesion to transport channels [53]. 

## 4. Conclusions

In this study, the effect of EDA amount on the structure and sieving performance of GO was studied by regulating interlayer nanochannels and adding different EDA amounts. EDA can form C–N covalent bonds via amidation with GO, which helps to inhibit the swelling of GO interlayer nanochannels. In the process of regulating layer spacing, it was found that an appropriate amount of EDA was beneficial to improving the mass transfer process of water molecules in interlayer nanochannels, whereas an excessive amount of EDA would lead to the dense structure of GO and improve the selectivity of Na_2_SO_4_ and NaCl. In addition, interlayer spacing was almost not increased. In conclusion, a stable interlayer nanomass transfer channel was constructed by embedding a high amount of EDA into the GO membrane, which is very important for GO to control ion transport as a nanofiltration membrane. 

## Figures and Tables

**Table 1:** XPS analysis of MCE/GO and MCE/GO/EDA-X membranes.

Sample	O/%	N/%	C/%	O/C
MCE/GO	32.14	-	67.86	0.47
MCE/GO/EDA-1	27.1	4.79	68.11	0.40
MCE/GO/EDA-3	24.72	4.9	70.38	0.35
MCE/GO/EDA-5	22.4	5.14	72.46	0.31
MCE/GO/EDA-7	21.89	5.63	72.48	0.30
MCE/GO/EDA-9	21.2	5.74	73.06	0.29

## Data Availability

The data presented in this study are available on request from the corresponding author.
